# Epidemiology of occupational injuries in Kerman province during 2012-2016

**DOI:** 10.5249/jivr.v14i1.1580

**Published:** 2022-01

**Authors:** Shiva Pouradeli, Mohsen Rezaeian, Vahid Rahmanian

**Affiliations:** ^*a*^ Occupational Environment Research Center, Medical School, Rafsanjan University of Medical Sciences.; ^*b*^ Social Determinants of Health Research Center, Institute for Futures Studies in Health, Kerman University of Medical Sciences, Kerman, Iran.; ^*c*^ Department of Epidemiology and Biostatistics, Occupational Environment Research Center, Medical School, Rafsan-jan University of Medical Sciences, Rafsanjan, Iran.; ^*d*^ Research Center for Social Determinants of Health, Jahrom University of Medical Sciences, Jahrom, Iran.

**Keywords:** Epidemiology, Injuries, Kerman Occupational injuries

## Abstract

**Background:**

According to the World Health Organization, occupational injuries are significant health issues globally that affect social lives and economic status. This study aimed to assess the situation of occupational injuries in the Kerman province.

**Methods:**

In this cross-sectional study, all occupational injuries registered in the Department of Cooperatives Labor and Social Welfare of Kerman Province were investigated during 2012-2016. Data were retrieved from an institutional database, including the official institutional software reports of Cooperatives Labor and Social Welfare occupational inspectors. The study used ArcGIS 10.3 software to prepare the geographical distribution of the cumulative incidence of occupational injuries on the map for each city. Data were analyzed using SPSS software.

**Results:**

A total of 2228 subjects with a mean age of 34 years and a mean work experience of 4.5years were injured during 5 years. 73.2% of them were married. 96.4% of them were Iranian, and 61.3% had insurance. The most number of injuries occurred in the construction industry, fractures being the typical outcome of the injuries. The most injured organ was the hands. Kerman has the highest number of injuries with 804 cases. Incidence rates ranged was 93 to 138 cases per 100,000 people in 5 years. The highest cumulative incidence rates of accidents occurred in Zarand and Kahnuj, respectively, in 5 years.

**Conclusions:**

Despite the decrease in occupational injuries in recent years, it is a severe problem in Kerman province. Occupational injuries cause irreparable damages to human resources, and it, directly and indirectly, imposes costs for the family and the government. Therefore, considering safety in occupational environments to prevent occupational injuries should be a priority in planning.

## Introduction

Occupational injuries are significant problems in high, middle, and low-income countries; nevertheless, efforts have reduced them. The World Health Organization (WHO) has described it as an epidemic in the public health field, and it is considered important health, economic and social risk factor. Globally, 31800 deaths due to occupational injuries and 2,022,000 deaths due to work-related diseases occurred annually.^1,2^

Occupational injuries increase direct and indirect costs for individuals and society by reducing efficiency and absenteeism in the workplace.^3^ Occupational injuries and economic disadvantage cause social anomalies.^4,5^ Previous studies have shown that environmental and personal factors can increase the incidence of occupational injuries.^2,6,7^

In recent years, technological advances, changes in work design, the use of personal protective equipment, and improvements in the culture of safety in organizations have led to significant improvements in workplace safety.^8^ Despite these advances and new rules to increase security in the workplace, many injuries happen at work, and many lives are lost in the process.^4^ Every organization and employer must create a safe environment for employees. Employers must regularly inspect the work environment for safety and notify authorities immediately if injuries occur.^9^

Working conditions for the larger number of the world's workers have lowest level of standards and guidelines collect by international agencies and occupational health and safety laws cover only about 10% of the population in low and middle income countries.^10^ “In order to properly implement Law and the technical protection criteria, Inspection Administration of Ministry of Labor and Social Affairs established in Iran to perform the following functions:

a. Supervision over enforcement of the regulations governing the working conditions

b. Supervision over proper enforcement of the provisions of the Labor Law, and by regulations and instructions pertaining to technical protection

c. Training matters pertaining to technical protection and guidance of workers, employers

d. Study and research with regard to the problems caused by the enforcement of technical protection regulations, and formulation of necessary proposals to amend the standards and directives 

e. Investigation of accidents arising out of work in the applicable workshops, and conducting general and statistical analysis of such cases”.^11^

Kerman is the largest and ninth most populous province in Iran, and its population was equal to 3,164,718 people according to the 2016 census. According to the latest national divisions, Kerman had 23 cities and 73 districts in 2016. Kerman is one of the most important and historical provinces in Iran. In southeastern Iran, this province is significant in industry, culture, politics, agriculture, higher education, and religion. According to the 2016 census, 915075 people older than 10 have worked in this province.^12,13^ Also, people live with many cultural and climate diversity in the province, affecting the job.^14^ Due to the large population of workers and various industries in Kerman, studying the information available regarding injuries in employment is the first and most crucial step to prevent injuries, improve safety, and increase the effectiveness and efficiency of workers and the workplaces. Thus, this study aimed to investigate the epidemiology of occupational injuries in social security insured workers over five years.

## Methods 

This cross-sectional study was approved by Ethics Committee of Kerman University of Medical Sciences. (Code IR.KMU.REC.1398.037) 

The population of this study included all insured workers who had occupational injuries in Kerman province for 5 years (2012-2016). The data included age, sex, education, marital status, work experience, nationality, insurance status, the previous experience of injury, training history, worker job, outcome of accident and injured organ by year, and cities of Kerman province. Data were retrieved from an institutional database, including the official institutional software reports of Cooperatives Labor and Social Welfare occupational inspectors. The data of some cities were collected in other cities. (Anar in Rafsanjan, Narmashir, Fahraj and Reigan in Bam, Anbarabad in Jiroft, Rudbar-e Jonubi, Qaleh Ganj and Faryab in Kahnuj and Orzuiyeh and Rabor in Baft). Then, the total number of insured workers was collected from the Social Security Organization of Kerman province between 2012-2016. In the next step, incidence rate of occupational injuries per 100000 people was calculated separately for each year and each city. ArcGIS 10.3 software was used to prepare the geographical distribution of the cumulative incidence of occupational injuries on the map for each city. Data were analyzed using SPSS software (version 20, SPSS Inc., Chicago, IL, USA). A significant level of 0.05 was considered.

(I)

$$ x=\frac{Number of occupational injuries in city and year}{Number of insured workers in city and year}*100000 $$

## Results

A total of 2,228 occupational injuries were recorded in the Department of Cooperatives Labor and Social Welfare of Kerman Province between 2012-2016. The mean age of the workers was 34.5 years, and the average work experience was 4.5 years. 97.9% were men, of them 73.2% were married, and 96.4% of the workers had Iranian nationality. 61.3% had social security insurance, and the rest had no insurance. 60.8% of them were never trained in occupational safety. 69.5% of them had no experience of injury in the past. (Table 1).

**Table 1:** Frequency of characteristics of the workers reporting occupational injuries, 2012 -2016.

Variables	Category	Frequency	Percent
**Sex**	Man	2181	97.9
	Woman	47	2.1
**Age**	<20	122	5.5
	20-30	916	41.1
	30-40	670	30.1
	40-50	334	15.0
	>50	186	8.3
**Status**	Single	597	26.8
	Married	1631	73.2
**Education**	Illiterate	379	17
	Diploma and less	1709	76.7
	Associate Degree or Bachelor	138	6.2
	Master's degree and more	2	0.1
**Experience**	<5	1430	64.2
	5-15	544	24.4
	15-25	182	8.2
	>25	72	3.2
**Nationality**	Iranian	2147	96.4
	Other	81	3.6
**Previous experience of injury**	Yes	92	2.7
	No	2362	69.5
**Covered by insurance**	Yes	1366	61.3
	No	862	37.7
**Training**	Yes	292	8.6
	No	1430	60.8

Construction jobs and other simple workers suffered the most number of injuries. The most common outcome of occupational injuries was fractures (877 cases), and the lowest outcome was that of poisoning (6 cases). In all occupations, fractures were significantly higher than the other injuries. (P-value = 0.0001) Hands and feet were the most injured organs. (Table 2).

**Table 2:** Frequency of information related to occupational injuries, 2012 -2016.

Variables	Category	Frequency	Percent
Worker job	Construction	812	36.4
	Simple workers	700	31.4
	Technical	311	14
	Service and office	128	5.7
	Mining	124	5.6
	Transportation	94	4.2
	Agricultural	28	1.3
	Others	31	1.4
Outcome of accident	Fracture	877	39.4
	Injury	438	19.7
	Death	263	11.8
	Maim	188	8.4
	Amputation	160	7.2
	Burns	69	3.1
	Poisoning	6	0.3
	Other - not mentioned	227	10.2
Injured organ	Hands	799	31
	Legs	519	20.1
	Head, Face and Neck	363	14.1
	Trunk and Back	239	9.3
	Nervous, Respiratory, Digestive or Circulatory system	37	1.4
	Other	613	23.8

The highest number of injuries occurred in 2012, Kerman with 804 cases, and Sirjan with 459 cases recorded to have the highest casualties among other cities.(Table 3)

**Table 3:** Frequency and Incidence(in 100000 people) of occupational injuries according to year and city.

City	2012			2013			2014		
	injury	Population	Incidence Rate	injury	Population	Incidence Rate	injury	Population	Incidence Rate
**Baft (Orzuiyeh and Rabor)**	24	14565	165	7	15207	46	4	15648	26
**Bardsir**	14	8813	159	18	9738	185	7	9525	73
**Bam (Fahraj and Narmashir) **	2	23062	9	4	23835	17	10	26409	38
**Jiroft (Anbarabad) **	30	17958	167	15	18487	81	15	19289	78
**Ravar**	0	7209	0	0	6951	0	0	6159	0
**Rafsanjan (Anar)**	66	52368	126	58	56170	103	35	57116	61
**Zarand**	47	21010	224	47	22626	208	52	24480	212
**Sirjan**	76	51966	146	72	56233	128	94	61404	153
**Shahr-e Babak**	14	12688	110	19	13823	137	20	14290	140
**Kerman**	206	142755	144	143	148547	96	117	156559	75
**Kuhbanan**	0	5138	0	0	5080	0	1	4943	20
**Kahnuj (Rudbar-e Jonubi, Qaleh Gan-jand Faryab) **	24	10499	229	27	10696	252	25	11296	221
**Manujan**	11	3809	289	11	3637	302	0	3596	0
**Total**	514	371840	138	421	391030	108	380	410714	93

**Chart 1:**
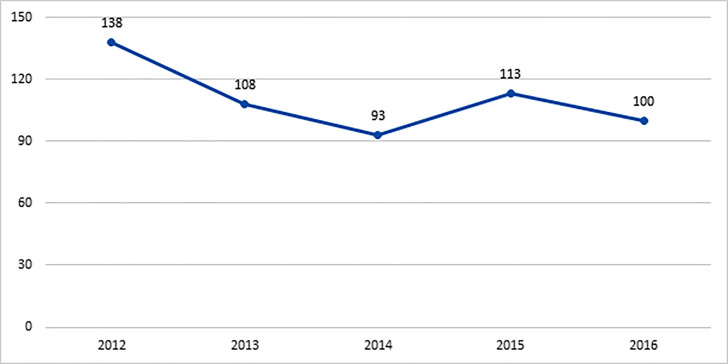
The incidence of occupational injuries per 100,000 insured people in period of 5 years.

The incidence of occupational injuries differed between 93-138 per 100,000 people in Kerman province in 5 years. (Chart 1)

**Figure 1:**
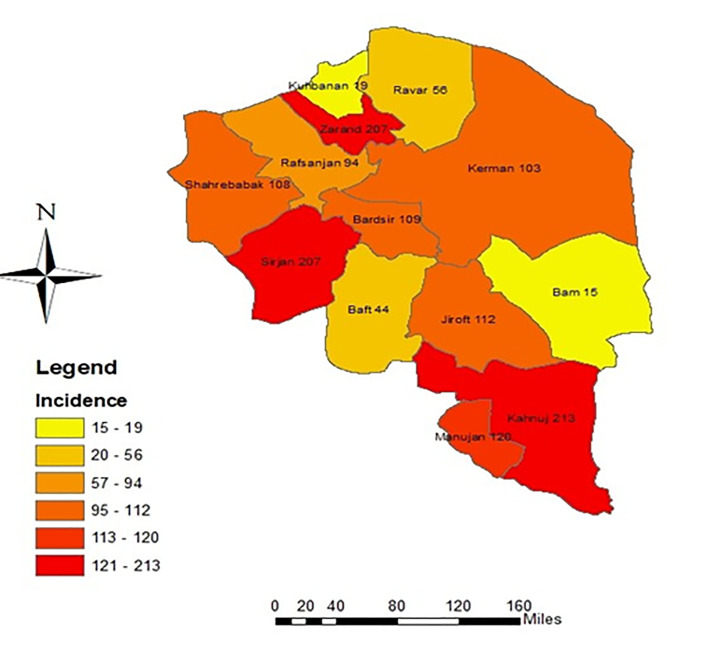
Cumulative incidence of occupational injuries in 5-years according to city.

The cumulative incidence of injuries has been higher in Zarand and Kahnuj in 5 years, respectively. (Figure 1)

## Discussion

According to this study, many workers are injured at work and some factors such as age, marriage, job, work experience can increase the risk of occupational injuries. Also, according to the previous studies, environmental and personal factors such as lack of awareness about safety,^6^ long working hours,^15^ hot weather,^16^ drug use, mental health problems, fatigue,^2^ being male, having a low age and low work experience,^17^ job type^18^ and even some social factors^7^ can increase the incidence of occupational injuries. The most important way to prevent occupational injuries is to educate about labor laws and safety in the work environment for workers.^19^

Men were more likely to have occupational injuries than women, consistent with previous studies.^5,6,19-24^ It could be due to men's more significant employment in high-risk jobs, though women are more cautious than men and pay more attention to safety issues.^24^ In this study, most occupational injuries are in the industrial and construction industry because women are less employed, leading to fewer injuries in women than in men.

In this study, the mean age of injured workers was 34 years. In Esmaeili's and AL-Abdallat's study, the mean age of the injured workers was 32 and 33 years, respectively.^5,20^ Also, in this study, most occupational injuries occurred to workers aged between 20 and 30 years. In Gholipour's study, most injures happened to those under 30 years of age.^21^ In the study of Ghods, most injuries happened to workers in the age group of 20 to 30 years, and in Dortaj's study, most injuries happened to workers in the age group of 25 to 30 years.^2,25^ The slight difference in high-risk groups in different studies is due to the different categories for age in each study, which yielded that most occupational injuries occurred with those aged 20-30 years. Khanzode's study found that workers under 25 years were at higher risk of occupational injuries, while injuries leading to death were higher in older people.^16^

Breslin's study found that workers under 35 years have more injuries, but the type of job plays a vital role in determining a solid relationship between age and injuries.^18^ Therefore, it is imperative to pay attention to the age of workers when they are employed in different jobs, and the type of job should be appropriate to the individual's ability. Occupational injuries will increase if workers' job is not appropriate in line with their ability.

More than half of the injuries occurred in workers who had less than 5 years of work experience in this study. Khanzode's study showed that work experience is one of the most critical factors in occupational injuries, and when work experience increases, the risk of injuries decreases.^16^ In Gholipour's study, less work experience caused more injuries.^21^

In the Esmaeili study, most injuries occurred in workers with less than 10 years of work experience.^5^ However, in Dortaj's study, the average work experience of workers was 13 years.^25^ According to the above results, workers' age and work experience are related, and younger workers with less work experience more suffer from occupational injuries.

In this study, almost three-quarters of injured workers were married, which is consistent with the studies by Quds, Esmaeili, and Grazier.^5,24,27^ Perhaps married workers were found to accept high-risk jobs regardless of the possible risks to cover their living expenses. But in Bakhtiari's study, single workers have more occupational injuries.^28^ Perhaps, this difference occurred because of cultural differences, especially in the south of the Kerman province with other provinces of Iran. In Kerman province, people married at a young age which increased the number of married workers with little work experience.

Workers in the construction industry had more injuries than those in other occupations, consistent with the Esmaili study in Rafsanjan and Doaraj study in Marvdasht.^5,25^ Also, in Grazier's study, construction jobs are considered high-risk jobs.^29^ In Tadesse's study, the most important cause of injuries in construction workers was lack of awareness regarding safety and unsuitable conditions in the workplace.^6^ In Hargreaves study, migrant workers works more in the agriculture; domestic, retail, and service sectors; construction and trade; and manufacturing and processing. The prevalence of at least one workplace injury, including falls from heights, fractures and dislocations, eye injuries, and cuts, was relatively common.^30^ So, workers must have sufficient knowledge about occupational safety laws. Therefore, monitoring the safety and health of workers and recognizing the risk factors of injuries in work is one of the essential tasks for the government, industry officials, and health professionals to prevent accidents and diseases in the workplace.^2^

In the study by Ghods, half of the injuries occurred in industrial occupations,^2^ which raises the probability that the construction industry in this city is less prosperous than in Kerman province. In Chau's study, sleep and smoking disorders (current smoker) were the reasons for falling in construction workers.^31^ In Halabi Study, most fall accidents occurred among the roofers, from heights less than 9.15 m, in new buildings and projects with low cost, between 10:00–12:00 and 13:00–15:00 and among older workers.^32^ Based on these results, creating appropriate and safe working conditions, paying attention to personality traits, and staff training can prevent injuries in these jobs. Fracture is the most common injury in this study, and hands were the most affected organ, consistent with the results of studies conducted by Esdaile and Dortaj.^5,25^ Also, In Bakhtiari's analysis, the most affected organs were the legs and then the hands.^28^ Due to the nature of the construction work, the probability of falling and injuring oneself is higher.

The incidence of occupational injuries has a different trend in Kerman province between 2012- 2016. It decreased in 2016 compared to 2012 in most cities. Perhaps attention to safety and compliance with regulations and oversight has improved in recent years.

The highest number of occupational injuries occurred in Kerman city, the capital of the province with a larger population. However, when the incidents of occupational injuries were investigated considering the population of each city, most injuries occurred in Zarand and Kahnuj. In general, the incidents of occupational injuries in most cities are higher than in Kerman city. Probably, this is the result of a lack of labor inspectors to monitor safety in the workplace. Therefore, the responsible organizations should have better plans to send labor inspectors from the center of the province to monitor workplace security in other cities.

More than half of the workers had social security insurance in this study, and the rest did not have any insurance. Many uninsured workers, especially non-Iranian workers, do not go to the insurance offices or Department of Cooperatives Labor and Social Welfare if they suffer from occupational injuries. The actual number of occupational injuries must be much higher than the data available with the Department. The results of the Shin study showed that considering private insurance, which pays more than public insurance, employers try to reduce more injuries and ensure safety for workers.^33^ Therefore, it seems that labor insurance in Iran should be reviewed and revised accordingly with stringent measures and actions.

## Conclusion

Despite occupational injuries decreasing in recent years, this issue is still a severe problem in Kerman province, especially in the construction industry. Most injuries occurred to people who were not trained and taught safety principles. Most of these injuries are preventable. Therefore, it is necessary to pay attention to personality traits and the ability of individuals in work, and adequate training of safety issues in the workplace. Also, increase periodic inspections of workplaces to enforce safety rules in workplaces.
